# Clinical and genetic characteristics of Chinese patients with cerebrotendinous xanthomatosis

**DOI:** 10.1186/s13023-019-1252-9

**Published:** 2019-12-03

**Authors:** Qing-Qing Tao, Yun Zhang, Hui-Xia Lin, Hai-Lin Dong, Wang Ni, Zhi-Ying Wu

**Affiliations:** Department of Neurology and Research Center of Neurology in Second Affiliated Hospital, and Key Laboratory of Medical Neurobiology of Zhejiang Province, Zhejiang University School of Medicine, 88 Jiefang Road, Hangzhou, People’s Republic of China

**Keywords:** Cerebrotendinous xanthomatosis, *CYP27A1*, Mutation, Clinical feature

## Abstract

**Background:**

Cerebrotendinous xanthomatosis (CTX) is a rare inborn lipid-storage disease caused by mutations in the sterol 27-hydroxylase (*CYP27A1*) gene with an autosomal recessive pattern of inheritance. To date, only 19 CTX patients from 16 families have been reported in the Chinese population.

**Results:**

Three novel likely pathogenic mutations (c.368_374delCCAGTAC, c.389 T > A and c.571C > T) and 7 previously reported pathogenic mutations (c.379C > T, c.435G > T, c.1016C > T, c.1214G > A, c.1263 + 1G > A, c.1420C > T and c.1435C > T) were identified. In addition, we summarized the genotypes and phenotypes of reported Chinese CTX patients. The most predominant mutations in *CYP27A1* were c.410G > A and c.379C > T, and the most common clinical manifestations were pyramidal signs, xanthomatosis, cerebellar ataxia, and cognitive impairment.

**Conclusion:**

Our study broadens the genetic and clinical spectrum of CTX and provides insightful information to help better diagnose and understand the disease.

## Introduction

Cerebrotendinous xanthomatosis (CTX) (OMIM: 213700) is a rare inborn lipid-storage disease, characterized by accumulation of cholestanol-containing xanthomas predominantly in tendons and the brain [1]. CTX is caused by mutations in the sterol 27-hydroxylase gene (*CYP27A1*) [2]. The human *CYP27A1* gene is located on chromosome 2 and contains 9 exons and encodes sterol 27-hydroxylase. Sterol 27-hydroxylase is a mitochondrial cytochrome P450 enzyme that plays a critical role in side-chain oxidation of cholesterol necessary for the synthesis of the bile acid [3–5]. The ability to convert cholesterol to bile acids is impaired in CTX patients, leading to the elevations of cholestanol and accumulation of cholesterol and cholestanol in multiple tissues, such as tendons, the central nervous system and lungs [6–8]. The common clinical presentations include infantile-onset chronic diarrhea, juvenile cataracts, progressive cognitive dysfunction and dementia, cerebellar ataxia, spasticity, osteoporosis, peripheral polyneuropathy and other atypical neurological symptoms [9–12]. However, the clinical manifestations of CTX can vary significantly even within the same family [13].

To date, over 100 variants in the *CYP27A1* gene and more than 300 CTX patients have been identified worldwide [14, 15]. In the Chinese population, only 19 patients from 16 families have been reported [16–27]. Here, we reported the genetic features and clinical findings of 6 unrelated Chinese patients with CTX and summarized the genotypes and phenotypes of all Chinese patients with CTX.

## Methods

### Subjects and clinical evaluation

Six pedigrees of CTX, including 6 patients and 12 family members, were collected from July 2015 to December 2018. The clinical evaluations and neurological examinations were performed by two senior neurologists. This study was approved by the Ethics Committee of Second Affiliated Hospital, Zhejiang University School of Medicine. Written informed consents were obtained from all the participants.

### Genetic testing of *CYP27A1*

Genomic DNA was extracted from peripheral blood samples using a commercial blood genomic extraction kit (Qiagen, Hilden, Germany). Polymerase chain reaction (PCR) was carried out to amplify all exons and flanking regions of *CYP27A1*. Direct Sanger sequencing was performed on an ABI 3500xl Dx Genetic Analyzer (Applied Biosystems, Foster City, CA, USA) as described previously [28]. The primers for *CYP27A1* were listed in Additional file 1: Table S1. The 1000 Genomes Project (https://www.ncbi.nlm.nih. gov/variation/tools/1000 genomes/) and the ExAC database (https://exac.broadinstitute.org/) were used to check the frequency of variants in the general population. Three software programs, including SIFT (http://sift.jcvi.org/), PolyPhen-2 (http://genetics.bwh.harvard.edu/pph2/) and Mutation Taster (http://www.mutationtaster.org/) were used to predict the possible protein functional changes caused by the variants.

### Literature review

We reviewed all of the CTX patients reported in the Chinese population from 1992 to April 31, 2019. Nineteen patients with integrated clinical information in 13 studies were included in our study [8, 14–18, 20–27]. The genotypes and phenotypes of Chinese CTX patients were summarized.

## Results

### Mutations identified in *CYP27A1*

Three novel variants including c.368_374delCCAGTAC, c.389 T > A (p.M130K) and c.571C > T (p.Q191*), and 7 previously reported pathogenic mutations (c.379C > T, c.435G > T, c.1016C > T, c.1214G > A, c.1263 + 1G > A, c.1420C > T and c.1435C > T) in *CYP27A1* (ClinVar database: https://www.ncbi.nlm.Nih.gov/clinvar/) were identified in 6 CTX families. The 3 novel variants were not found in the 1000 Genomes Project and the ExAC databases. Additionally, they were not found in our targeted next-generation sequencing (NGS) database that covered *CYP27A1*, which contained 800 Chinese subjects without CTX. According to the guidelines provided by the American College of Medical Genetics (ACMG), c.368_374delCCAGTAC (1 piece of very strong pathogenic evidence and 3 pieces of moderate pathogenic evidence), c.389 T > A and c.571C > T (3 pieces of moderate pathogenic evidence and 2 pieces of supporting pathogenic evidence) were classified as likely pathogenic mutations [29].

### Clinical features of six CTX patients

**Fig. 1:**
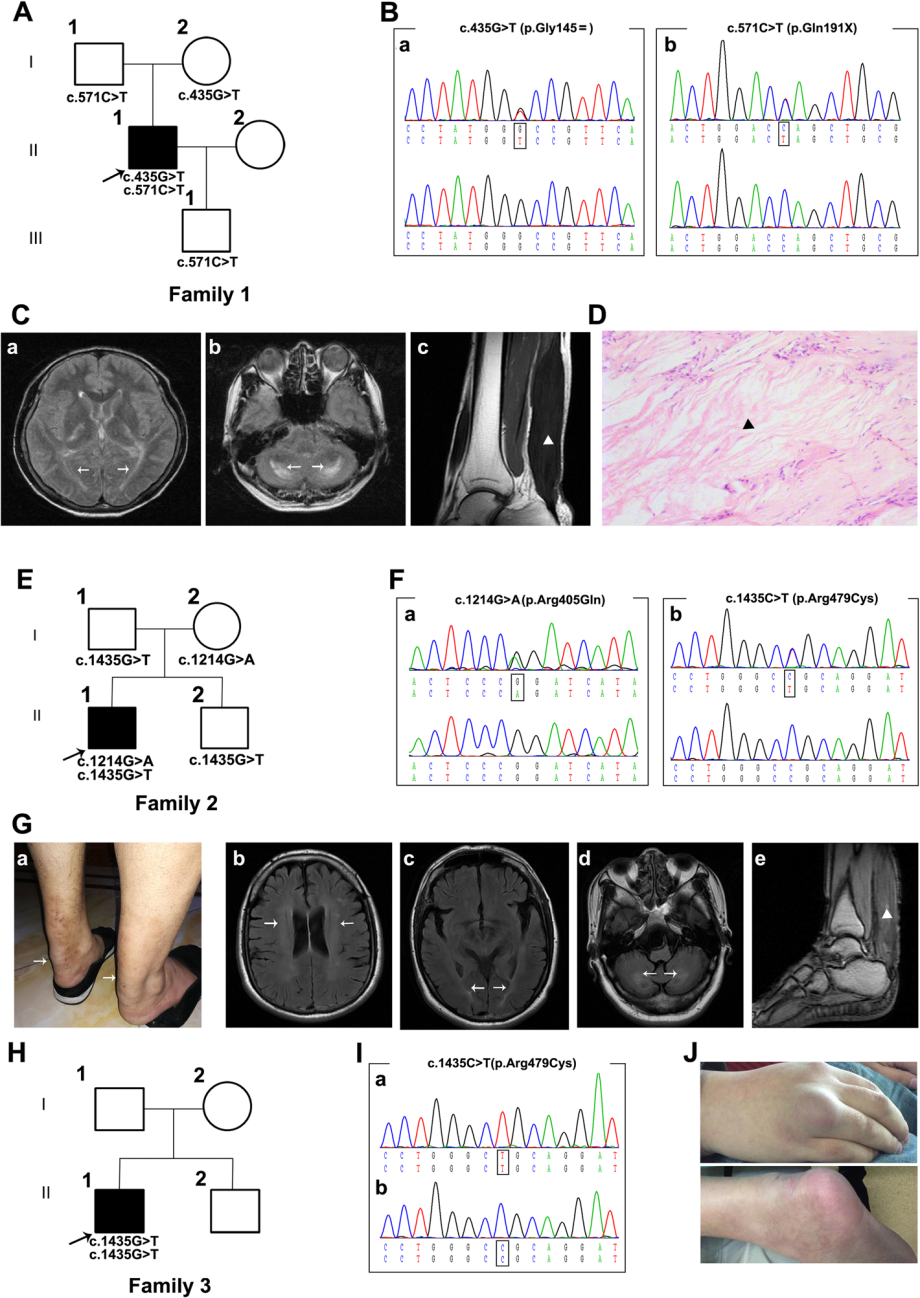
Pedigree charts and clinical findings of Family 1–3**. A, E, H.** Pedigree charts of 3 Chinese CTX families, *Squares* indicate males; *circles* indicate females; *black* symbols indicate affected individuals; the *arrow* indicates the proband. **B.** The chromatogram of the *CYP27A1* variants (a.435G > T and b.c.571C > T) identified in Family 1**. C.** Hyperintense signals in bilateral cerebella and posterior cerebral white matter fibers of proband in Family 1 (a and b); Sagittal proton density-weighted image shows fusiform thickening of the Achilles tendon (c) (*marked with arrow*). **D.** HE staining of the tendon masses reveals dispersed lipid crystal clefts. 100×. **F.** The chromatogram of *CYP27A1* variants (c.1214G > A and c.1435C > T) identified in Family 2 (a and b) (*marked with triangle*). **G.** Enlargement of the Achilles tendons of proband in Family 2 (a); Hyperintense signals in bilateral cerebella, lateral ventricle and posterior cerebral white matter fibers of proband in Family 2 (b, c and d); Hyperintense signal on T1-weighted images of proband in Family 2 (e) (marked with arrow and triangle). **I.** The chromatogram of *CYP27A1* variant (c.1435G > T) identified in Family 3. **J.** Subcutaneous masses of proband in Family 3

The proband in Family 1 **(**Fig. 1A**)** was found to carry one novel likely pathogenic mutation (c.571C > T, p.Q191*) and one previously recognized mutation (c.435G > T, p.G145=) **(**Fig. 1B**)**. It is worth mentioning that the synonymous mutation c.435G > T (p.G145=) was previously reported as a pathogenic mutation that causes alternative pre-mRNA splicing of *CYP27A1* [30]. The proband in Family 1 was a 45-year-old male presenting with a 7-year history of slowly progressive gait disturbance and clumsy movement. He noticed xanthomas in bilateral Achilles tendons at 36 years old, and a surgical operation was performed to remove the xanthomas two years later. He was diagnosed with CTX and received simvastatin (20 mg per day) treatment for approximately one year. However, the above symptoms gradually worsened. Symptoms originated with mild stiffness in the neck and right upper extremity two years ago, followed by slurred speech and occasional depression. In addition, gait disturbance became more serious with significant unsteadiness when walking downstairs. The above symptoms developed gradually during the next two years, and now the patient cannot walk without auxiliary equipment. On examination, he had bilateral enlargement of the Achilles tendons and subcutaneous masses. Neurologic examinations revealed dysarthria and gait ataxia. Cognitive function was normal with a Mini-Mental State Examination (MMSE) score of 28. The muscle strength of the limbs was 5/5. Increased tendon reflexes were observed. Bilateral Hoffman signs and Babinski signs were positive. He was unable to touch the tip of his nose with his index finger, wipe one palm alternately with the palm and dorsum of the other hand, and slide the heel of one foot down the shin of the other leg. The plasma cholestanol concentration was not tested because there is a lack of proper test methods for plasma cholestanol levels in most hospitals in China. Electromyography (EMG) showed multiple motor sensory demyelinating peripheral neuropathies. Brain magnetic resonance imaging (MRI) demonstrated hyperintense signals in the bilateral cerebella and posterior cerebral white matter fibers **(**Fig. 1C**)**. Histological examination of the paraffin section of the tendon showed lipid crystal clefts in hematoxylin-eosin (H-E) staining **(**Fig. 1D**).**

The proband from Family 2 **(**Fig. 1E**)** carried two reported pathogenic missense mutations, c.1214G > A (p.R405Q) and c.1435C > T (p.R479C) **(**Fig. 1F**)**. He was a 40-year-old male admitted to our hospital with a chief complaint of a 3-year history of slowly progressive gait disturbance. He noticed xanthomas in his bilateral Achilles tendons one year ago, and a surgical operation was performed to remove the xanthomas in a local hospital. In the last four months, his gait disturbance developed gradually. He denied symptoms of cognitive impairment, sight loss or numbness. Physical examination showed bilateral mild swelling of the Achilles tendons. Neurological examinations showed that the muscle strength of the right limbs was 4/5 and 5/5 in the left limbs. Bilateral Babinski signs were positive. She swayed slightly when she touched the tip of her nose with index finger. It is difficult for her to wipe her palm quickly and place the heel on the knee. Brain MRI indicated cerebellar atrophy and hyperintense signals in the bilateral cerebella and posterior cerebral white matter fibers **(**Fig. 1G**)**. An MRI scan of the ankle showed hyperintense and hypertrophy of the gastrocnemius and peroneus longus **(**Fig. 1G**)**.

The proband from Family 3 **(**Fig. 1H**)** carried a pathogenic homozygous mutation of c.1435C > T (p.R479C) **(**Fig. 1I**)***.* He was a 30-year-old male admitted to our hospital presenting with a 24-year history of cognitive impairment and a 15-year history of gait disturbance. He developed transient loss of consciousness and epileptic seizure attack at 6 years old and presented cognitive impairment in the next year. At 15 years old, progressively unsteady gait developed, causing falls, especially when running, followed by gradual weakness and progressive spasm and paresis in legs. At 22 years old, he developed bilateral blurred vision and was diagnosed with cataracts. Vision was recovered after the surgical operation was performed four years later. He had enlargement of tendons and subcutaneous masses in hands **(**Fig. 1J**)**. Neurological examinations showed mild cognitive impairment with an MMSE score of 21. The muscle strength of the upper limbs was normal, while it was 4/5 in the lower limbs. Bilateral Hoffman signs and Babinski signs were positive. Bilateral tendon reflexes increased in the lower limbs.

**Fig. 2:**
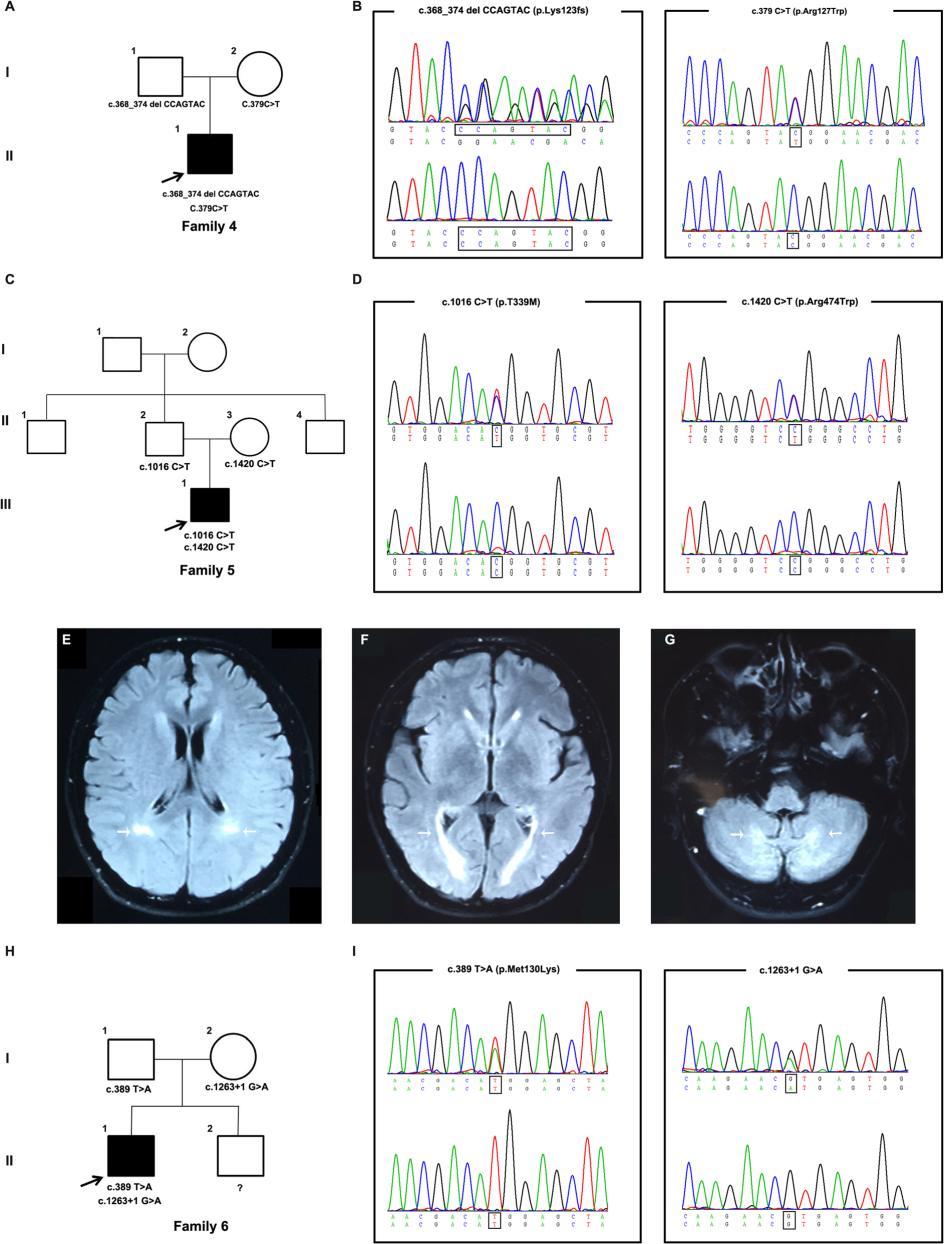
Pedigree charts and clinical findings of Family 4–6**. a, c, h** Pedigree charts of 3 Chinese CTX families, Squares indicate males; *circles* indicate females; *black* symbols indicate affected individuals; the *arrow* indicates the proband. **b** The chromatogram of the *CYP27A1* variants (c.368_374delCCAGTAC and c.379C > T) identified in Family 4**. d** The chromatogram of *CYP27A1* variants (c.1016C > T and c.1420C > T) identified in Family 2. **e-g** Hyperintense signals in bilateral cerebella and posterior cerebral white matter fibers of proband in Family 5 (*marked with arrow*). **i** The chromatogram of *CYP27A1* variant (c.389 T > A and c.1263 + 1G > A) identified in Family 6

The proband from Family 4 **(**Fig. 2a**)** was identified to have one novel likely pathogenic mutation (c.368_374delCCAGTAC, p.L123 fs) and one previously reported pathogenic mutation (c.379C > T, p.R127W) **(**Fig. 2b**)***.* He was a 32-year-old male admitted to our hospital with a chief complaint of a 20-year history of gait disturbance. He developed progressively unsteady gait at 12 years old, followed by gradually spasm and paresis in legs. On examination, he had a short stature and bilateral pes cavus deformity. No enlargement of tendons was noticed. Neurological examinations showed that the muscle strength of the lower limbs was 4/5. He was unable to touch the tip of his nose with his index finger. Bilateral quadriceps and gastrocnemius muscle atrophy were found. Bilateral Hoffman signs and Babinski signs were positive. Bilateral tendon reflexes increased in all limbs (4+).

The proband from Family 5 **(**Fig. 2c**)** was found to carry two reported pathogenic missense mutations, c.1016C > T (p.T339 M) and c.1420C > T (p.R474W) **(**Fig. 2d**)***.* He was a 24-year-old male who presented with a 20-year history of slowly progressive gait disturbance. Unsteady gait first appeared at 4 years old. One year ago, his gait disturbance became worse. On examination, he had scoliosis and bilateral pes cavus deformity. Neurological examinations showed mild cognitive impairment with an MMSE score of 22. Mild atrophy of the thenar and interosseous muscles was found in the right hand. Bilateral Hoffman signs and Babinski signs were positive. The tendon reflexes of the extremities were symmetrically increased (4+). Brain MRI demonstrated hyperintense signals in the bilateral periventricular white matter **(**Fig. 2e-g**)**.

The proband in Family 6 **(**Fig. 2h**)** carried one novel likely pathogenic mutation (c.389 T > A, p.M130K) and one previously reported pathogenic mutation (c.1263 + 1G > A) **(**Fig. 2i**)***.* He was a 27-year-old male who came to our clinic with a chief complaint of a half-year history of gait disturbance. He denied sight loss or numbness. No tendons enlargement was noticed. Neurological examinations showed bilateral tendon reflexes increased in all four limbs (3+). Bilateral Babinski signs were positive. The main clinical findings of these 6 patients are summarized in Table 1.

**Table 1:** Clinical features of six patients with cerebrotendinous xanthomatosis

Variable	Patient1	Patient2	Patient3	Patient4	Patient5	Patient6
CYP27A1 mutation	c.435G > T	c.1214G > A	c.1435C > T	c.368_374delCCAGTAC	c.1016C > T	c.389 T > A
	c.571C > T	c.1435C > T	c.1435C > T	c.379C > T	c.1420C > T	c.1263 + 1G > A
Gender	Male	Male	Male	Male	Male	Male
Age	45	40	30	32	24	27
Age of diagnosis	38	40	30	32	24	27
Diarrhoea	–	–	+	–	–	–
Tendon xanthomas	+	+	+	–	–	–
Cataracts	–	–	+	–	–	–
Epilepsy	–	–	+	–	–	–
Cognitive impairment	–	–	+	–	+	–
Pyramidal signs	+	+	+	+	+	+
Cerebellar signs	+	+	+	–	+	–
EEG abnormality	+	+	NP	+	+	+
Dentate nuclei lesions	+	+	NP	–	+	–

NP: Not present

### Genotypes and phenotypes of Chinese CTX patients

**Fig. 3:**
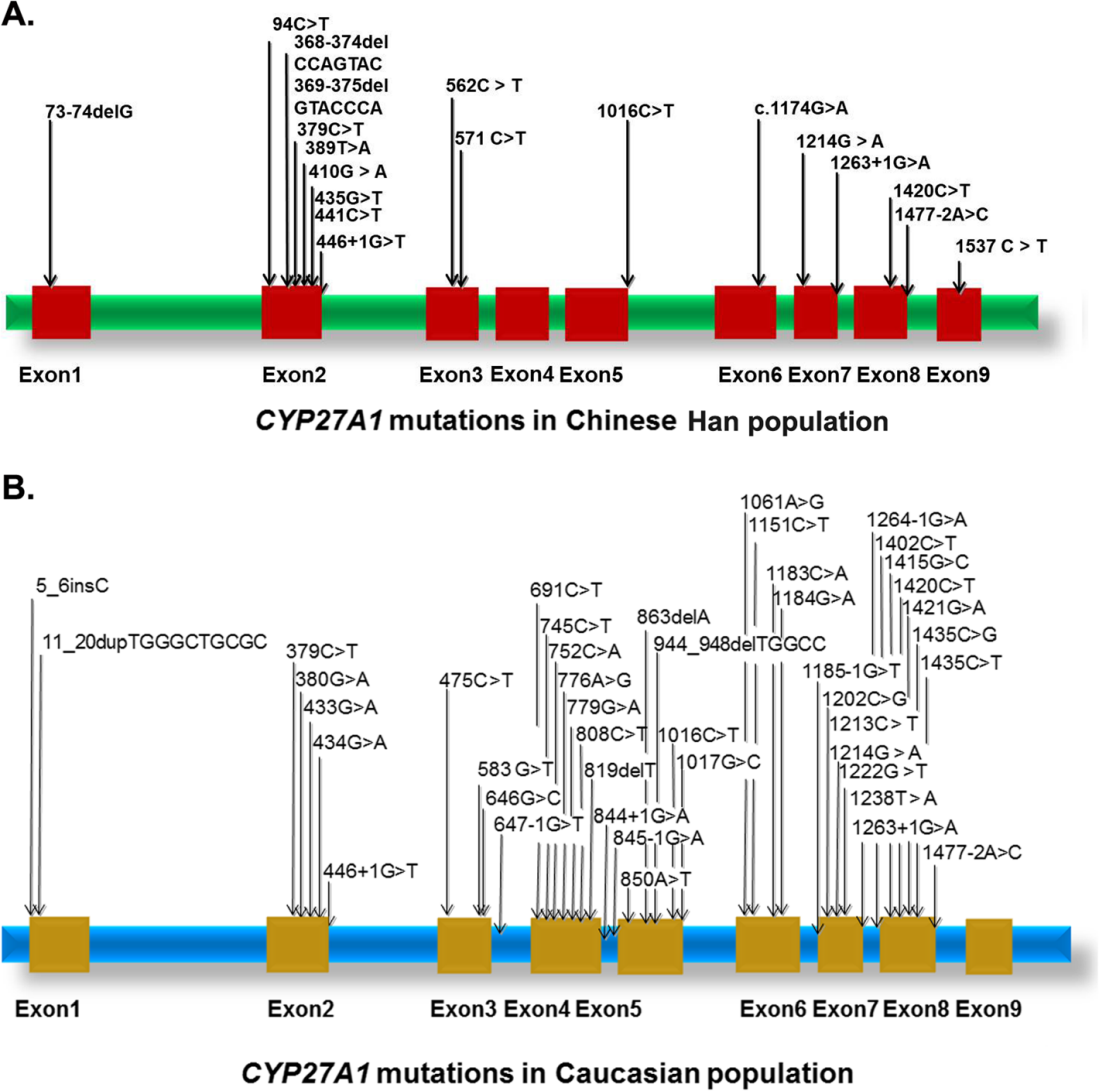
The spectrum of *CYP27A1* pathogenic mutations in Chinese and Caucasian populations was depicted according to ClinVar database (https://www.ncbi.nlm.nih.gov/clinvar/) **a** *CYP27A1* pathogenic mutations in the Chinese population. **b** *CYP27A1* pathogenic mutations in the Caucasian population

We reviewed all of the previous CTX patients reported in the Chinese population and found that the most frequent mutations in *CYP27A1* were c.410G > A (p.R137Q, 22.7%), c.379C > T (p.R127W, 18.2%), c.1435C > T (p.R479C, 9%) and c.305delC (p.P102Lfs, 9%) **(**Table 2**)**. Combined with our study and a previous study^16^, the most frequent clinical manifestations of CTX patients in the Chinese population were pyramidal signs (88.5%), xanthomatosis (84.6%), cerebellar ataxia (57.7%), cognitive impairment (57.7%), cataracts (38.5%), and peripheral neuropathy (30.8%), which were quite different from those in the Caucasian population **(**Table 3**)**. Moreover, the spectrum of *CYP27A1* mutations in the Caucasian population differed from that in the Chinese Han population **(**Fig. 3**).**

**Table 2:** Clinical and genetic features of patients with cerebrotendinous xanthomatosis in the Chinese population

Family	Case	Geographical distribution	Mutation 1	Mutation 2	AAO	AAE	Clinical symptoms and signs	Reference
1	1	Mainland SE	p.Q191X	p.G145=	36	38	Xanthoma; Cerebellar ataxia; Pyramidal signs; Peripheral neuropathy	This study
2	2	Mainland SE	p.R405Q	p.R479C	37	40	Xanthoma; Cerebellar ataxia; Pyramidal signs; Peripheral neuropathy	This study
3	3	Mainland SE	p.R479C	p.R479C	6	30	Xanthoma; Cognitive impairment; Pyramidal signs; Cataracts; Chronic diarrhea; Epilepsy	This study
4	4	Mainland SE	p.K123 fs	p.R127W	12	32	Pyramidal signs;Peripheral neuropathy	This study
5	5	Mainland SE	p.T339 M	p.R474W	4	24	Pyramidal signs;Cerebellar ataxia; Cognitive impairment; Peripheral neuropathy	This study
6	6	Mainland SE	p.M130K	c.1263 + 1G > A	26	27	Pyramidal signs;Peripheral neuropathy	This study
7	7	Mainland SE	p.R513C	c.1477-2A > C	33	48	Xanthoma; Cerebellar ataxia; Pyramidal signs; Cognitive impairment; Peripheral neuropathy	Chen et al.2017 [16]
7	8	Mainland SE	p.R513C	c.1477-2A > C	NG	43	Xanthoma; Pyramidal signs; Cognitive impairment	Chen et al.2017 [16]
8	9	Mainland SE	p.R137Q	p.R137Q	30	35	Xanthoma; Pyramidal signs	Chen et al.2017 [16]
9	10	Mainland SE	p.R137Q	p.R127W	42	44	Xanthoma; Pyramidal signs	Chen et al.2017 [16]
10	11	Mainland SE	p.R188X	p.R405Q	33	37	Xanthoma; Cerebellar ataxia; Pyramidal signs; Cognitive impairment	Chen et al.2017 [16]
11	12	Mainland SE	p.R127W	p.E392K	8	27	Xanthoma; Cerebellar ataxia; Pyramidal signs; Cognitive impairment; Cataracts	Zhang et al.2016 [22]
12	13	Mainland S	c.446 + 1G > T	p.T339 M	14	14	Xanthoma; Cerebellar ataxia; Cognitive impairment; Cataracts	Zhong et al.2014 [26]
13	14	Mainland SE	p.T339 M	p.T339 M	NG	36	Xanthoma; Cerebellar ataxia; Pyramidal signs; Cognitive impairment;	Wei et al.2012 [25]
14	15	Mainland N	c.73-74delG	c.369-375delGTACCCA	7	36	Xanthoma; Cerebellar ataxia; Pyramidal signs; Cataracts	Tian et al.2011 [18]
15	16	Taiwan	c.205-206delC	p.R104W	NG	42	Xanthoma; Cerebellar ataxia; Pyramidal signs; Cognitive impairment; Peripheral neuropathy	Chen et al.2011 [20]
16	17	Mainland N	p.R127W	p.R474W	20	42	Xanthoma	Wang et al.2007 [19]
17	18	Taiwan	p.P102Lfs	p.P102Lfs	6	42	Xanthoma; Pyramidal signs	Wang et al.2006 [21]
18	19	Hong kong	c.1185-1G > T	p.R372Q	7	48	Xanthoma; Cerebellar ataxia; Pyramidal signs; Cognitive impairment; Cataracts	Mak et al. 2004 [23]
18	20	Hong kong	c.1185-1G > T	p.R372Q	NG	44	Xanthoma; Pyramidal signs	Mak et al. 2004 [23]
18	21	Hong kong	c.1185-1G > T	p.R372Q	NG	50	Xanthoma; Pyramidal signs; Cognitive impairment; Cataracts	Mak et al. 2004 [23]
19	22	Taiwan	c.1263 + 1G > A	p.R127W	NG	NG	Xanthoma; Cerebellar ataxia; Pyramidal signs; Cognitive impairment; Cataracts; Peripheral neuropathy	Lee et al.2002 [27]
20	23	Hong kong	Unknown	Unknown	16	34	Xanthoma; Cerebellar ataxia; Pyramidal signs; Cognitive impairment; Cataracts	Ko et al.2001 [24]
21	24	NG	p.G472A	p.G472A	NG	NG	Xanthoma; Cerebellar ataxia; Pyramidal signs; Cognitive impairment; Cataracts; Peripheral neuropathy	Verrips et al. 2000 [14]
22	25	Taiwan	Unknown	Unknown	NG	31	Xanthoma; Cerebellar ataxia; Pyramidal signs; Cognitive impairment; Cataracts	Chang et al.1992 [17]

*SE* Southeast, *S* South, *N* North, *NG* Not given, *AAO* Age at onset, *AAE* Age at examination

**Table 3:** Clinical features of patients with CTX in different populations

Clinical phenotypes	Chinese population (*n* = 25)	Caucasian population (Spanish n = 25)	*p*-value
Pyramidal signs	88.5%	92.0%	*P* = 0.157
Xanthomatosis	84.6%	56.0%	*P* < 0.01
Cerebellar ataxia	57.7%	76.0%	*P* < 0.05
Cognitive impairment	57.7%	Not given	
Cataracts	38.5%	92.0%	P < 0.01
Peripheral neuropathy	30.8%	64.0%	P < 0.05
Chronic diarrhea	3.8%	92.0%	P < 0.01
Epilepsy	3.8%	32.0%	P < 0.01

## Discussion

CTX is a rare sterol storage disease caused by mutations in *CYP27A1* with an autosomal recessive pattern of inheritance [31]. Since the first CTX patient was reported in 1937, more than 300 patients have been reported worldwide [32]^,^ and 19 patients have been reported in the Chinese Han population [16]. There is no consensus on the prevalence of CTX, with an estimated rate of < 5/100,000 worldwide [33]. Currently, 108 variants of the *CYP27A1* gene have been reported, and over 50 variants were considered pathogenic or likely pathogenic according to the Human Gene Mutation Database (HGMD). As CTX is a potentially treatable disease, early diagnosis and treatment are critical to improve the prognosis of CTX. However, diagnosis usually has a delay of several years [34]. Summarizing the genetic and clinical characteristics to help early diagnosis and treatment from a clinical perspective has great significance.

In our study, we reported 6 Chinese families with CTX. The diagnosis of CTX was confirmed by genetic sequencing of the *CYP27A1* gene. Three novel likely pathogenic mutations (c.368_374delCCAGTAC, c.389 T > A, c.571C > T) and 7 previously reported pathogenic mutations (c.379C > T, c.435G > T, c.1016C > T, c.1214G > A, c.1263 + 1G > A, c.1420C > T and c.1435C > T) in *CYP27A1* were identified in our study. According to a recent nationwide survey on CTX in Japan, the most frequent mutations in the *CYP27A1* gene were c.1214G > A (p.R405Q, 31.6%), c.1421G > A (p.R474Q, 26.3%), and c.435G > T (p.Gly145=, 15.8%) [35]. In the Chinese population, we found that the most frequent mutations in the *CYP27A1* gene were c.410G > A (p.R137Q, 22.7%), c.379C > T (p.R127W, 18.2%), and c.1435C > T (p.R479C, 9%). The most frequent mutations reported in the Japanese population, such as c.1214G > A (p.R405Q, 31.6%) and c.435G > T (p.G145=, 15.8%) were also found in the Chinese population. Many more CTX patients have been reported in the Caucasian population than in the Chinese Han population. However, the spectrum of *CYP27A1* mutations in the Caucasian population differed from that in the Chinese Han population. The most frequent mutations in *CYP27A1* were located in exon 2 (50%) in the Chinese Han population and in the region from exon 4 to exon 8 (75%) in the Caucasian population.

Combined with our study and a previously reported study [16], the most frequent clinical manifestations of CTX patients in the Chinese population were pyramidal signs, xanthomatosis, cerebellar ataxia, cognitive impairment, cataracts, and peripheral neuropathy. In our study, we first reported that a CTX patient had initial symptoms of epileptic seizure attack, expanding the clinical spectrum of CTX in the Chinese population. The most common CTX symptoms in the Japanese population were tendon xanthoma, followed by spastic paraplegia, cognitive dysfunction, cataract, ataxia, and epilepsy [35]. In a study performed in the Spanish population containing 25 CTX patients, the most common clinical manifestations were chronic diarrhea, cataracts, pyramidal signs, cerebellar ataxia, peripheral neuropathy and xanthomatosis [36].

The genetic and clinical characteristics differed greatly between the Chinese and Caucasian populations. Several reasons need to be considered. First, as CTX is a rare disease, the sample size is relatively small in most studies in the Chinese population, multicenter studies with large samples may help to clearly identify the characteristics of CTX in the population. Second, genetic background may be one of the major reasons for the differences in CTX genotypes and phenotypes between the Chinese and Caucasian populations. In addition, most of the hospitals in China have no proper test methods for plasma cholestanol level, leading to most of the CTX patients not being diagnosed until tendon xanthomas were observed. However, the emerging development of target next-generation sequencing will help better diagnose the disease.

## Conclusions

In conclusion, we reported 6 CTX families of Chinese Han origin. Three novel likely pathogenic mutations including c.368_374delCCAGTAC, c.389 T > A, c.571C > T in CYP27A1 were identified. In addition, we compared the genetic and clinical features of CTX between Chinese and Caucasian population. In the Chinese population, the most predominant mutations in the *CYP27A1* gene were c.410G > A (p.R137Q) and c.379C > T (p.R127W), the most frequent clinical manifestations were pyramidal signs, xanthomatosis, cerebellar ataxia, and cognitive impairment.

## Supplementary information

**Additional file 1 Table S1.** Primer sequences of *CYP27A1. (DOCX 14 kb)*

## Data Availability

All data generated or analyzed during this study are included in this published article.

## Abbreviations

CTX: Cerebrotendinous xanthomatosis
LDL: Low-density lipoprotein
MMSE: Mini-Mental State Examination
MRI: Magnetic resonance imaging
NGS: Next-generation sequencing
PCR: Polymerase chain reaction;
