# Supplementary material for: Clinical and genetic characteristics of Chinese patients with cerebrotendinous xanthomatosis
Source: Orphanet J Rare Dis. 2019 Dec 3;14:282. doi: 10.1186/s13023-019-1252-9 (PMC6892200; doi:10.1186/s13023-019-1252-9)
Supplement: Supplementary file 1 — Additional file 1 Table S1. Primer sequences of CYP27A1. (DOCX 14 kb) [file 13023_2019_1252_MOESM1_ESM.docx]

| **Supplementary Table 1. Primer sequences of *CYP27A1*** | | |
| --- | --- | --- |
| **Primer** |  | **Primer sequence (5'to 3')** |
| Exon 1 | 1F | ACTCAGCACTCGACCCAAAGG |
|  | 1R | CCAGTTTATGACCATAGCCTC |
| Exon 2 | 2F | GCCCAGCTCATTTGCTCTTG |
|  | 2R | CAACTGCTCTCTAGACCATC |
| Exon 3 | 3F | CTAAGATTCTGTCACTTGAG |
|  | 3R | AGCAAATAGCTGAGTAGTGC |
| Exon 4 | 4F | TTGGAAGGTACCCTTGCTGG |
|  | 4R | TCCGACAACTGGTAGCATGA |
| Exon 5 | 5F | GACTCCAGGTCTGTGCATCA |
|  | 5R | ATGAAGGTCGGGATCAGGTGAG |
| Exon 6 | 6F | GCTAGGCTAGTGGCAAATTC |
|  | 6R | CCCACCTGTCTTCACTTCAG |
| Exon 7 | 7F | AAGTGAAGACAGGTGGGCTG |
|  | 7R | GGCAGAACACAAACTGGGTC |
| Exon 8 | 8F | TGAAGTTGATGGCTTCCTC |
|  | 8R | ATTGTGTGTTTGCCATCCAC |
| Exon 9 | 9F | ATGCAGCTACTCCTCGCAAGG |
|  | 9R | AAGGAGTTCCTCCCACCTCTC |
